# Integrative Single-Cell Analysis of Cardiomyopathy Identifies Differences in Cell Stemness and Transcriptional Regulatory Networks among Fibroblast Subpopulations

**DOI:** 10.1155/2024/3131633

**Published:** 2024-05-18

**Authors:** Wenyang Nie, Zhijie Zhao, Yuhang Liu, Youcao Wang, Jingwen Zhang, Ying Hu, Yang Liu, Yong Wang, Zhen Wang

**Affiliations:** ^1^Department of Cardiovascular Diseases, Affiliated Hospital of Shandong University of Traditional Chinese Medicine, 16369 Jing 10 Rd, Jinan 250000, China; ^2^First Clinical Medical College, Shandong University of Traditional Chinese Medicine, 16369 Jing 10 Rd, Jinan 250000, China; ^3^Department of Plastic and Reconstructive Surgery, Shanghai 9th People's Hospital, School of Medicine, Shanghai Jiao Tong University, 639 Zhi Zao Ju Rd, Shanghai 200011, China; ^4^Shanghai Jiao Tong University School of Medicine, 227 Chongqing South Rd, Shanghai 200025, China; ^5^School of Acupuncture, Moxibustion and Tuina, Shandong University of Traditional Chinese Medicine, 4655 University Rd, Jinan 250355, China

## Abstract

**Background:**

Cardiomyopathy encompasses a broad spectrum of diseases affecting myocardial tissue, characterized clinically by abnormalities in cardiac structure, heart failure, and/or arrhythmias. Clinically heterogeneous, major types include dilated cardiomyopathy (DCM), hypertrophic cardiomyopathy (HCM), restrictive cardiomyopathy (RM), ischemic cardiomyopathy (ICM), among which DCM is more prevalent, while ICM exhibits higher incidence and mortality rates. Myocardial injury during cardiomyopathy progression may lead to myocardial fibrosis. Failure to intervene early and inhibit the process of myocardial fibrosis may culminate in heart failure. Cardiac fibroblasts constitute crucial cellular components determining the extent and quality of myocardial fibrosis, with various subpopulations exerting diverse roles in cardiomyopathy progression. Despite this, understanding of the cellular plasticity and transcriptional regulatory networks of cardiac fibroblasts in cardiomyopathy remains limited. Therefore, in this study, we conducted comprehensive single-cell analysis of cardiac fibroblasts in cardiomyopathy to explore differences in cellular plasticity and transcriptional regulatory networks among fibroblast subpopulations, with the aim of providing as many useful references as possible for the diagnosis, prognosis, and treatment of cardiomyopathy.

**Materials and Methods:**

Cells with mitochondrial gene expression comprising >20% of total expressed genes were excluded. Differential expression genes (DEGs) and stemness genes within cardiac fibroblast subpopulations were subjected to Gene Ontology (GO) analysis of biological processes (BP) and AUCell analysis. Monocle software was employed to analyze the pseudo-temporal trajectory of cardiac fibroblasts in cardiomyopathy. Additionally, the Python package SCENIC was utilized to assess enrichment of transcription factors and activity of regulators within cardiac fibroblast subpopulations in cardiomyopathy.

**Results:**

Following batch effect correction, 179,927 cells were clustered into 32 clusters, designated as T_NK cells, endothelial cells, myeloid cells, fibroblasts, pericytes, SMCs, CMs, proliferating cells, EndoCs, and EPCs. Among them, 8148 fibroblasts were further subdivided into 4 subpopulations, namely C0 THBS4+ Fibroblasts, C1 LINC01133+ Fibroblasts, C2 FGF7+ Fibroblasts, and C3 AGT + Fibroblasts. Results from GO_BP and AUCell analyses suggest that C3 AGT + Fibroblasts may be associated with immune response activation, protein transport, and myocardial contractile function, correlating with disease progression in cardiomyopathy. Transcription factor enrichment analysis indicates that FOS is the most significant TF in C3 AGT + Fibroblasts, also associated with the M1 module, possibly implicated in protein hydrolysis, intracellular DNA replication, and cell proliferation. Moreover, correlation analysis of transcriptional regulatory activity between fibroblast subpopulations reveals a more pronounced heterogeneity within C3 AGT + Fibroblasts in cardiomyopathy.

**Conclusion:**

C3 AGT + Fibroblasts exhibit increased sensitivity towards adverse outcomes in cardiomyopathy, such as myocardial fibrosis and impaired cardiac contractile function, compared to other cardiac fibroblast subpopulations. The differential cellular plasticity and transcriptional regulatory activity between C3 AGT + Fibroblasts and other subgroups offer new perspectives for targeting fibroblast subpopulation activity to treat cardiomyopathy. Additionally, stemness genes EPAS1 and MYC, along with the regulator FOS, may play roles in modulating the biological processes of cardiac fibroblasts in cardiomyopathy.

## 1. Introduction

Cardiomyopathies are a large group of diseases that involve myocardial tissue and are characterized clinically by structural abnormalities of the heart, heart failure, and/or arrhythmias. There is great clinical heterogeneity and diversity. Cardiomyopathy is primarily classified into ischemic cardiomyopathy and non-ischemic cardiomyopathy. Distinguishing between ischemic and non-ischemic cardiomyopathy is often pivotal in cardiac management [1]. Cardiomyopathy was classified by WHO in 1995 into dilated cardiomyopathy (DCM), hypertrophic cardiomyopathy (HCM), restrictive cardiomyopathy (RM), ischemic cardiomyopathy (ICM), etc [2]. DCM is characterized by diffuse systolic dysfunction of the left ventricle and dilatation of left ventricular chambers [3], and it is the most common form of cardiac disease in heart failure and sudden cardiac death. one of the most common causes of sudden cardiac death [4]. As DCM is a genetically heterogeneous disease [5], familial DCM (FDCM) inheritance is predominantly based on an autosomal dominant pattern of inheritance [6]. In addition to this, there is also acquired DCM due to nutritional deficiencies, infections, alcohol, and administration of cardiotoxic drugs [7, 8]. RM has a variety of etiologic factors, such as endocardial processes, infiltrative processes, radiation, drug exposure, and mutations in the myonodal apparatus, which increase myocardial stiffness and impaired relaxation, leading to pulmonary hypertension and heart failure [9]. RM is not as common as DCM, so research on RM is often overlooked, and the lack of effective treatment leads to the worst prognosis for RCM [10]. ICM is a common cardiovascular disease caused by chronic myocardial ischemia [11], and it is the most prevalent cause of heart failure in developed countries. Despite the optimization of the management of coronary artery disease and the development of percutaneous coronary intervention (PCI), which has improved the survival of patients with acute myocardial infarction (AMI), the morbidity and mortality rates of ICM are still high and remain a public health burden [12, 13].

The process of cardiomyopathy inevitably involves myocardial injury, which may lead to myocardial fibrosis, especially DCM, ultimately predisposing the heart to arrhythmias and heart failure [14]. A study employing bioinformatics analysis identified key hub genes and associated molecules potentially highly correlated with dilated cardiomyopathy (DCM), among which four hub genes (COL3A1, COL1A2, LUM, and THBS4) showed significant enrichment in the fibrosis pathway [15]. In addition, cardiac remodeling, including myocardial fibrosis and likewise pathophysiological manifestations such as cardiac hypertrophy and angiogenesis, is a way of altering myocardial structure in order to compensate for cardiac insufficiency, which has been significantly associated with the development of heart failure [16]. However, despite all current clarity that inhibition of cardiac fibrosis remains a key therapeutic direction to stop the transition from cardiomyopathy to heart failure, no study has yet demonstrated the discovery of effective molecular targets [17]. Cardiomyocytes are not the only cell population associated with the heart; the heart also consists of endothelial cells, smooth muscle cells, and cardiac fibroblasts, among others [18, 19]. Cardiac fibroblasts are the key cellular component that determines the extent and quality of myocardial fibrosis. Fibroblasts include different subtypes based on embryonic origin, tissue location, and function, and it has been shown that their activity is controlled to some extent by pro-inflammatory cytokines (such as TNF-*α*, IL-1, IL-17), which promote phenotypic switching and altered extracellular matrix (ECM) production [20–22]. At the same time, activated fibroblasts generate a positive feedback loop that produces chemokines that attract inflammatory cells, further increasing inflammation and potentially enhancing the fibrotic process [21]. Cardiac fibroblasts play various roles in the damaged heart [23], however, their specific function in cardiomyopathy is unknown. Hirofumi et al. demonstrated that cardiac fibroblasts play a pathogenic role in idiopathic RM [24]. Mizuki et al. demonstrated that cardiac fibroblasts from patients with idiopathic RM impaired the diastolic function of healthy cardiomyocytes [25].

And various transcriptional regulators and signaling pathways are involved in the pathogenesis of cardiac remodeling [26]. However, there are fewer studies targeting transcriptional regulatory factors among fibroblast subtypes associated with cardiomyopathy and their activities. Meanwhile, the expression of stemness genes and metabolic pathways in cardiomyopathy-associated fibroblast subtypes may be closely related to the progression of cardiomyopathy.

Therefore, in this study, we attempted to visualize cardiomyopathic fibroblasts by scRNA-seq, to gain insight into the heterogeneity of cardiomyopathic fibroblasts, and to analyze fibroblast transcriptional regulator activities. To provide a reference for future possibilities to stop the progression of cardiomyopathy to heart failure and the outcome of myocardial fibrosis from cardiac fibroblasts as an entry point.

## 2. Materials and Methods

### 2.1. Single-Cell Source Data Acquisition and Processing

The scRNA-seq data from cardiomyopathy fibroblasts were downloaded from GSE145154 via the Gene Expression Omnibus database (https://www.ncbi.nlm.nih.gov/geo/). The 10X genomics data for each sample were subsequently loaded into R software (4.1.3) using the Seurat software package (v4.1.1). The DoubletFinder program (v2.0.3) was used to remove potentially bimodal cells [27], filter low-quality cells, and control the cell quality to the following ranges: 300 < nFeature < 7500, 500 < nCount < 100000, cells meeting the above ranges will be retained and used in the next step of the data analysis, and the expression of mitochondrial genes in a cell is less than 20% of the total number. In this study, we excluded low-quality cells with less than 500 or more than 6000 identified genes. Because we used data from publicly accessible databases, this study did not require ethical approval.

### 2.2. Clustering and Cell Type Identification by scRNA-seq

To perform the natural logarithmic transformation, we used the log(*x* + 1) method to calculate the gene expression in each cell as a fraction of genes multiplied by 10,000. the normalized expression matrix was subsequently used to identify the top 2,000 highly variable genes (HVGs), which were then normalized [28]. These genes were subsequently analyzed in PCA. We used harmony's method to remove the batch effect between samples and selected the top 30 PCs for downscaling and clustering. After the dimensionality reduction clustering the results were projected onto a 2D map using the UMAP method for the next step of cell type identification [29, 30]. We obtained the cell markers of the relevant cells by referring to previous literature as well as according to the CellMarker database (https://xteam.xbio.top/CellMarker/), annotated the cell clusters by cell markers, identified different cell types, and observed the distribution and proportion of different cell types. In addition, in order to further investigate the heterogeneity of cardiomyopathy fibroblasts, we then reclustered the cardiomyopathy fibroblasts and subsequently labeled each cardiomyopathy fibroblast subpopulation according to its unique genes.

### 2.3. Enrichment Analysis of Differentially Expressed Genes and AUCell Analysis

We used the “FindAllMarkers” function based on the Wilcoxon rank-sum test with default parameters to distinguish differentially expressed genes (DEGs), as well as differentially stemness genes, in each cell type of cardiomyopathy and each subpopulation of fibroblasts. Genes expressed in more than 25% of cells in clusters with logFC values greater than 0.25 were also selected. To further understand the function of each cell type, we performed enrichment analysis of DEGs genes for each cell type and fibroblast subpopulation in cardiomyopathy using clusterProfiler [31, 32]. The pathways for each cell type were derived from the gene ontology (GO) biological process (BP) [33–37].

AUCell [38] is a method for identifying cells with active genes in single-cell RNA-seq data. the input to AUCell is a gene set, and the output is the “activity” of that gene set in each cell type.

### 2.4. Pseudo-Temporal Ordering of Fibroblast Subpopulations

We used the Monocle2 software toolkit (version 2.22.0) to analyze pseudotemporal trajectories of cardiomyopathy fibroblasts. By using pseudo-temporal profiles of scRNA-seq data, Monocle was able to identify cellular changes that occur during differentiation of cardiomyopathy fibroblasts. DDRTree technology was used to perform FindVairableFeatures and downscaling. Arrange cardiomyopathy fibroblasts according to pseudotime and observe the development of each subpopulation of cells under pseudotemporal trajectories.

### 2.5. SCENIC Analysis

SCENIC is a tool that utilizes scRNA-seq data to reconstruct gene regulatory networks while being able to identify stable cell states. In this study, we used the pySCENIC (version 0.10.0) package in Python (version 3.7) and its default parameters to generate a matrix of AUCell values to assess transcription factor enrichment and regulator activity [39].

## 3. Results

### 3.1. Cell Type and Heterogeneity in Cardiomyopathy

**Figure 1:**
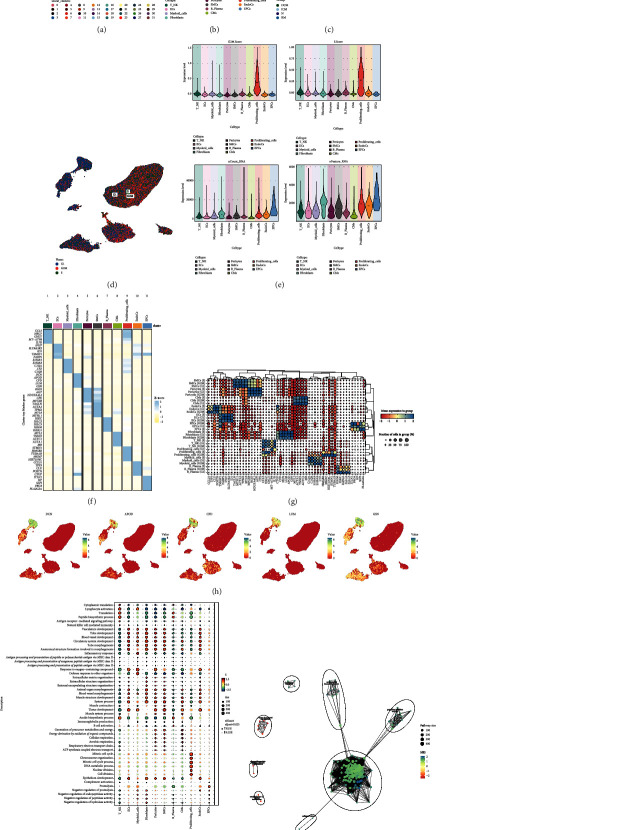
Cell type identification and enrichment analysis of cardiomyopathy. (a) 179927 high-quality cells were categorized into 32 clusters and the distribution of the 32 clusters was demonstrated using UMAP plots. (b) UMAP plot showed the distribution of 11 cell types. (c) UMAP plot demonstrated the distribution of 4 groups in 11 cell types. (d) UMAP plot demonstrated the distribution of different cell cycles in 11 cell types. (e) Violin plot demonstrated G2M. Score, S.Score, nCount RNA, nFeature RNA in 11 cell types. (f) Heatmap showed the differential expression of Top5 maker genes for 11 cell types. (g) Bubble plot demonstrated the differential expression of maker genes for each of the 11 cell types when they are in different cell cycles. (h) UMAP plot demonstrated the differential expression distribution of Top5 maker genes in cardiomyopathy fibroblasts. (i) Bubble plot demonstrated the GO_BP enrichment results of 11 cell types. (j) Enrichment network graph demonstrated the enrichment of all differential genes among subpopulations of cardiomyopathy fibroblasts.

After correction by batch results, we grouped 179,927 high-quality cells into 32 cell clusters (Figure 1(a)). And the retained high-quality cells after screening were classified into 11 cell types by specific marker genes, which were T_NK cells, ECs, myeloid cells, fibroblasts, Pericytes, SMCs, CMs, proliferating cells, EndoCs, EPCs (Figure 1(b)). Next, we showed the distribution of different groups and cell cycles of cardiomyopathies in UMAP diagrams (Figures 1(c) and 1(d)). According to the violin diagram in Figure 1(e), we learned that proliferating cells had the highest G2M score and S score, and all other cell types were lower. And for nCount_RNA, EPCs had the highest, followed by proliferating cells, Fibroblasts and EndoCs. Similarly, EPCs had the highest nFeature_RNA, followed by proliferating cells, fibroblasts, and EndoCs. To fully characterize the various cell types of cardiomyopathy, we relied on the differential expression of specific differential genes for each cell type, and the Top5 marker genes for the various cell types were demonstrated with a heat map (Figure 1(f)). In more detail, we further presented the differential genes specifically expressed by the 11 cell types at their respective different cell cycles, as shown in the bubble diagram in Figure 1(g). The results are largely consistent with the heatmap. Subsequently, we visualized the expression distribution of Top5 marker genes highly expressed in cardiomyopathic fibroblasts more visually by UMAP plots (Figure 1(h)). According to GO_BP analysis, the main enrichment results of 11 cell types were shown in bubble plots (Figure 1(i)), in which fibroblasts were mainly enriched in vasculature development, tube development, blood vessel development, circulatory system development, tube morphogenesis, anatomical structure formation involved in morphogenesis, System process etc. Subsequently, we individually enriched all differential genes among fibroblasts, and the enrichment network diagram showed that the pathways enriched for differential genes in fibroblasts were activation of immune response, peptidase inhibitor activity, collagen trimer, cholesterol transport (Figure 1(j)).

### 3.2. Annotation and Enrichment Analysis of Cardiomyopathy Fibroblast Subpopulations

**Figure 2:**
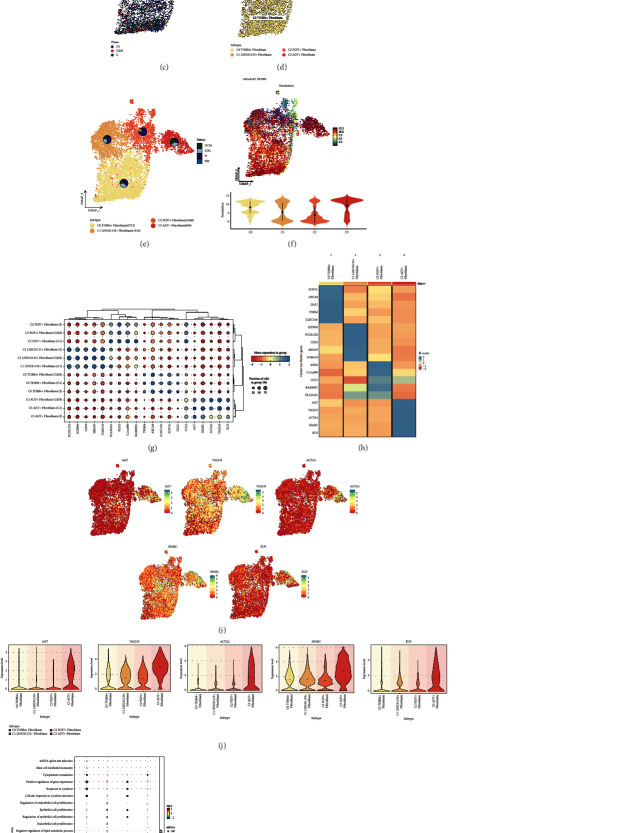
Identification and enrichment analysis of cardiomyopathy fibroblast subpopulations. (a) The 8148 fibroblasts were divided into 4 clusters and the distribution of the 4 clusters was demonstrated using UMAP plot. (b) UMAP plot showed the distribution of the 4 groups in all fibroblasts. (c) UMAP plot demonstrated the distribution of different cell cycles in all fibroblasts. (d) All fibroblasts were categorized into 4 cellular subgroups based on specific marker genes, and the distribution was demonstrated using UMAP plots. (e) UMAP shots showed the proportion of each group in the four fibroblast subsets. (f) UMAP plots and violin plots demonstrated the results of the proposed time series of fibroblast subpopulations. (g) Bubble plots demonstrated the differential expression of maker genes when each of the four fibroblast subpopulations is in different cell cycles. (h) Heatmap showed the differential expression of Top5 maker genes for each of the 4 fibroblast subpopulations. (i) UMAP plot demonstrated the expression distribution of Top5 maker genes in C3 AGT + Fibroblasts. (j) Violin plot demonstrated the expression comparison of Top5 maker genes of C3 AGT + Fibroblast. (k) Bubble plot demonstrated the GO_BP enrichment results of 4 fibroblast subpopulations. (l) Enrichment network graph demonstrated the enrichment of all differential genes within C3 AGT + Fibroblast.

We selected fibroblasts from cardiomyopathies for analysis and obtained 8148 high-quality fibroblasts after quality control and removal of batch effects, and first classified them into 4 cell clusters (Figure 2(a)). Subsequently, we visualized the distribution of the 4 groups of cardiomyopathies and different cell cycles in fibroblasts using UMAP plots (Figures 2(b) and 2(c)). Based on the differential expression of specific differential genes (THBS4, LINC01133, FGF7, AGT), we classified fibroblasts into 4 subgroups, the distribution of which is shown in Figure 2(d). The highest percentage of N was found in C1 LINC01133+ Fibroblasts and C2 FGF7+ Fibroblasts, the higher percentage of ICM and N in C0 THBS4+ Fibroblasts, the highest percentage of N in C1 LINC01133+ Fibroblasts, and the highest percentage of N in C2 FGF7+ Fibroblasts had the highest percentage of N, and the percentage of ICM and DCM was higher in C3 AGT + Fibroblasts (Figure 2(e)). To further investigate the heterogeneity of cardiomyopathic fibroblasts, we analyzed the 4 fibroblast subpopulations using monocle, and the results were depicted by UMAP plots and violin plots (Figure 2(f)). The UMAP plots demonstrated the distribution of pseudotimes in the 4 fibroblast subpopulations. As shown by the violin plots, C2 FGF7+ Fibroblasts were located at the onset of pseudotime and had the lowest degree of differentiation, and C3 AGT + Fibroblasts were located at the end of pseudotime and had the highest degree of differentiation. Meanwhile, C3 AGT + Fibroblasts had the highest percentage of G2M phase, active DNA replication and division, and high cell proliferation intensity. Therefore, C3 AGT + Fibroblasts may be associated with the progression of cardiomyopathy deterioration. For the accuracy of cardiomyopathy fibroblast identification, we showed the differential genes expressed by the 4 fibroblast subpopulations at their respective different cell cycles in bubble plots (Figure 2(g)). And the expression of Top5 marker genes of the 4 fibroblast subpopulations was depicted by heatmap, and the results were largely consistent with the bubble plots (Figure 2(h)). We selected the Top5 marker genes of C3 AGT + Fibroblasts and visually depicted their distribution among subpopulations by UMAP plot (Figure 2(i)). To clarify that five genes AGT, TAGLN, ACTA2, SPARC, ELN were specifically highly expressed in C3 AGT + Fibroblasts, it was demonstrated by violin plots (Figure 2(j)). The results of CO_BP enrichment analysis of the four fibroblast subpopulations were shown by bubble plots (Figure 2(k)), in which C3 AGT + Fibroblasts were mainly enriched in cytoplasmic translation, peptide biosynthetic process, actin filament-based process, translation, muscle contraction, peptide metabolic. Similarly, we enriched all the differential genes of C3 AGT + Fibroblasts, and we learned from the enrichment network diagram that the pathway in which the differential genes of C3 AGT + Fibroblasts were mainly enriched was multicellular organism development (Figure 2(l)).

### 3.3. The Degree of Activity of Oxidative Phosphorylation Metabolic Pathway between Fibroblasts Was Significant

**Figure 3:**
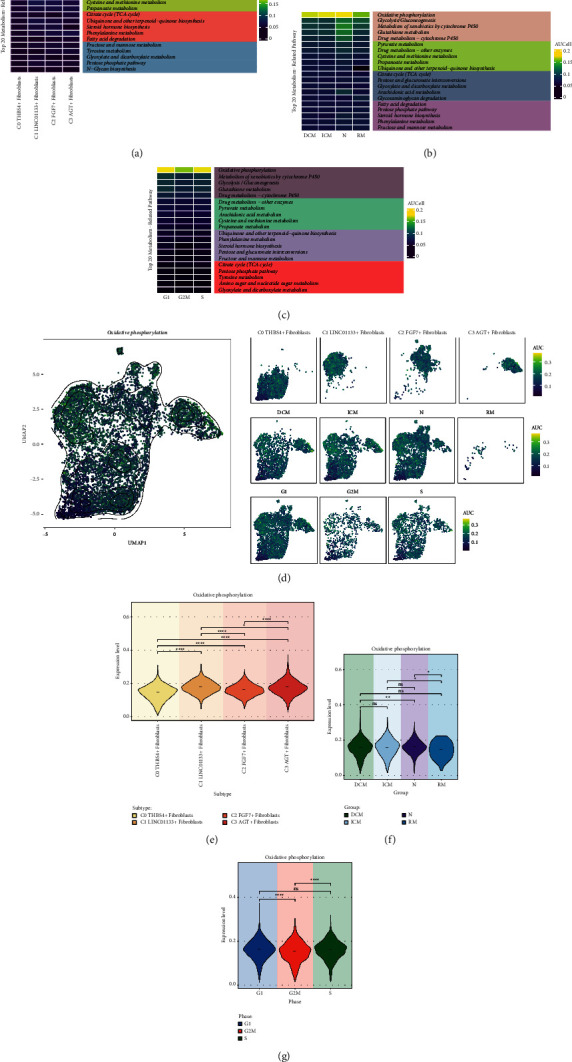
Metabolic pathways associated with cardiomyopathy fibroblast subpopulations. (a) Heatmap demonstrated the AUCell scores of Top20 metabolism-related pathways for 4 fibroblast subpopulations. (b) Heatmap demonstrated the AUCell scores of Top20 metabolism-related pathways for four groups. (c) Heatmap demonstrated the AUCell scores of Top20 metabolism-related pathway for different cell cycles. (d) UMAP plot and faceted graph demonstrated the distribution of Oxidative phosphorylation in 4 fibroblast subpopulations, 4 groups and different cell cycles with high and low AUCell scores. (e) Violin plots demonstrated the difference in the expression level of Oxidative phosphorylation in the 4 fibroblast subpopulations. (f) Violin plot showed the difference in the expression level of Oxidative phosphorylation in 4 groups. (g) Violin plot demonstrated the expression level differences of Oxidative phosphorylation in different cell cycles. ^*∗*^*p* < 0.05, ^*∗∗*^*p* < 0.01, ^*∗∗∗*^*p* < 0.001, ^*∗∗∗∗*^*p* < 0.0001, ns indicates no statistical difference.

To further investigate the metabolism of cardiomyopathic fibroblasts and increase the possibility of understanding the progression associated with cardiomyopathy. We presented the AUCell score values of the Top20 metabolism-related pathway of the 4 fibroblast subpopulations in a heat map (Figure 3(a)). Similarly, we also put the AUCell score values of Top20 Metabolism-Related Pathways for different groups and different cell cycles on display (Figures 3(b) and 3(c)), and the metabolism-related pathways with the highest AUCell scores were all oxidative phosphorylation. To visualize the distribution of oxidative phosphorylation among fibroblasts, we presented a UMAP plot (Figure 3(d)). From this, we learned the distribution of AUCell scores of oxidative phosphorylation across 4 fibroblast subpopulations, 4 groups and different cell cycle. Next, we used violin plots to precisely compare the expression levels of oxidative phosphorylation. Thus, we found that it was higher in C1 LINC01133+ Fibroblasts and C3 AGT + Fibroblasts (Figure 3(e)), a bit higher in ICM and N (Figure 3(f)), and higher in G1 phase and S phase (Figure 3(g)). This result was consistent with the heat map results.

### 3.4. Glycolysis/Gluconeogenesis Is Equally Significant in C3 AGT + Fibroblasts

**Figure 4:**
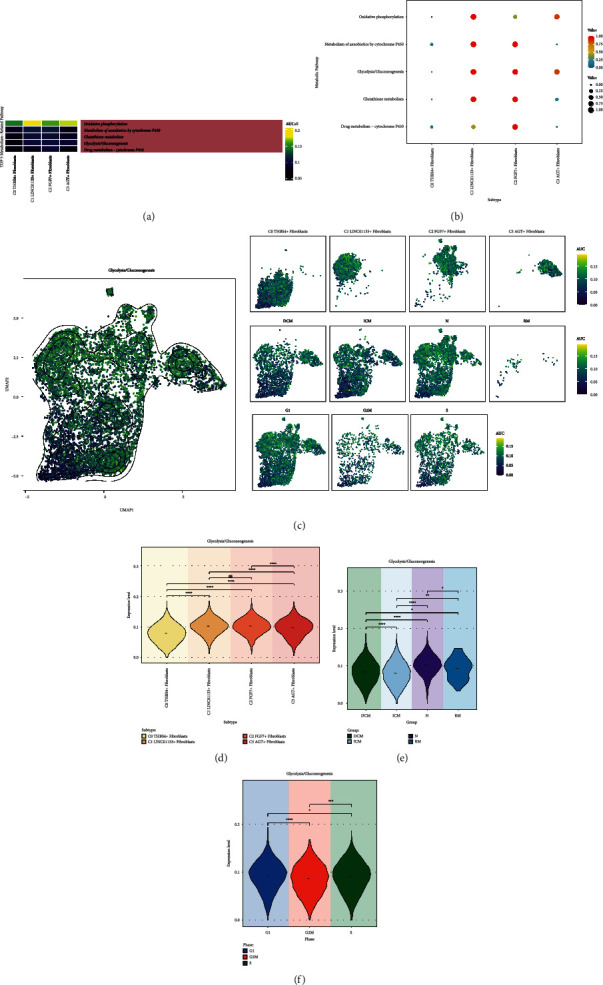
Glycolysis/glycolysis metabolic pathway. (a) Heatmap demonstrated the AUCell scores of Top5 metabolism-related pathways for four fibroblast subpopulations. (b) Bubble plot demonstrated the expression of the 5 metabolism pathways in the 4 fibroblast subpopulations. (c) UMAP plots and faceted plots demonstrated the distribution of high and low AUCell scores of glycolysis/gluconeogenesis in 4 fibroblast subpopulations, 4 groups and different cell cycles. (d) Violin plot showed the difference in expression levels of glycolysis/gluconeogenesis in 4 fibroblast subpopulations. (e) Violin plot showed the difference in expression levels of glycolysis/gluconeogenesis in 4 groups. (f) Violin plots demonstrated the expression level differences of glycolysis/gluconeogenesis in different cell cycles. ^*∗*^*p* < 0.05, ^*∗∗*^*p* < 0.01, ^*∗∗∗*^*p* < 0.001, ^*∗∗∗∗*^*p* < 0.0001, ns indicates no statistical difference.

We again presented the AUCell score values of Top5 Metabolism-Related Pathway for the four fibroblast subpopulations in a heat map (Figure 4(a)). We found that Glycolysis/Gluconeogenesis was more significant in C3 AGT + Fibroblasts, except for Oxidative phosphorylation. Similarly, we used bubble plots to show oxidative phosphorylation, metabolism of xenobiotics by cytochrome P450, glycolysis/gluconeogenesis, glutathione metabolism, drug metabolism-cytochrome P450 expression in four fibroblast subpopulations (Figure 4(b)). The results were consistent with the heat map that glycolysis/gluconeogenesis was the metabolic pathway ranked as the second most highly expressed in C3 AGT + Fibroblasts. To visualize the distribution of glycolysis/gluconeogenesis among fibroblasts, we demonstrated it with a UMAP plot (Figure 4(c)). From this, we learned the distribution of AUCell scores of glycolysis/gluconeogenesis among four fibroblast subpopulations, four groups and different cell cycle. Then, we used violin plots to precisely compare the expression levels of glycolysis/gluconeogenesis. Thus we found that it was higher in C1 LINC01133+ Fibroblasts, C2 FGF7+ Fibroblasts, C3 AGT + Fibroblasts (Figure 4(d)), a little bit higher in N and RM (Figure 4(e)), and higher in G1 phase and S phase (Figure 4(f)). This result is consistent with the heat map results.

### 3.5. Differential Expression of Stemness Genes in Fibroblast Subpopulations

**Figure 5:**
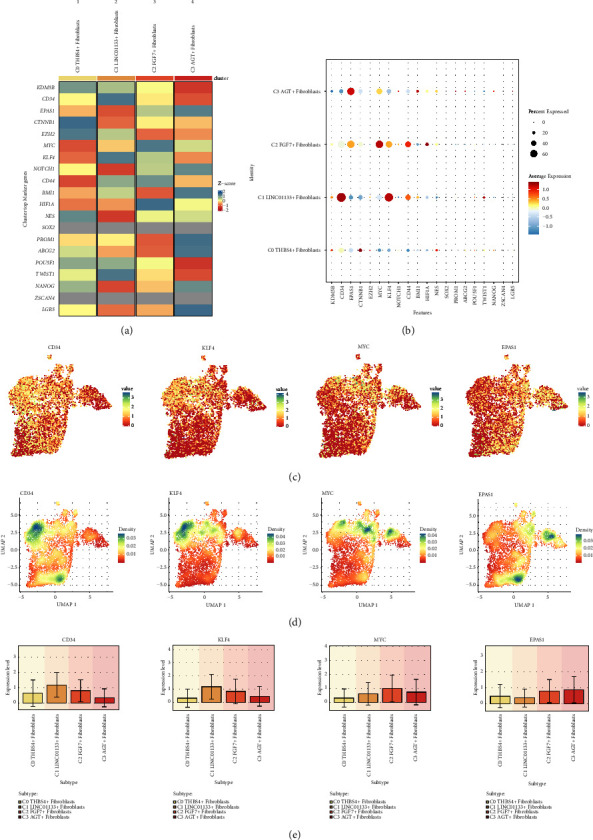
Visualization of stemness genes in cardiomyopathy fibroblast subpopulations. (a) Heatmap demonstrated the Z-score of differential stemness genes of the four fibroblast subpopulations. (b) Bubble plot demonstrated the average expression levels of differential stemness genes of the 4 fibroblast subpopulations. (c) UMAP plot demonstrated the distribution of the expression of the 4 stemness genes CD34, KLF4, MYC, EPAS1. (d) UMAP plot demonstrated the distribution of the densities of the 4 stemness genes CD34, KLF4, MYC, EPAS1. (e) Bar graph demonstrated the comparative expression levels of 4 stemness genes CD34, KLF4, MYC, EPAS1 in 4 fibroblast subpopulations.

We investigated the stemness gene expression of fibroblast subpopulations to understand the stemness characteristics of cardiomyopathic fibroblasts and the retardation or escape of cellular senescence, and thus provide some possible gene targets for stopping the continuous progression of cardiomyopathy. Therefore, we first demonstrated the top marker stemness genes of the 4 fibroblast subpopulations with heatmaps (Figure 5(a)). The Z-score of CTNNB1, EZH2 was higher in C0 THBS4+ Fibroblasts, and the Z-score of CD34, KLF4, TWIST1 was higher in C1 LINC01133 + Fibroblasts, Z-score of HIF1A, MYC, CD44 was higher in C2 FGF7+ Fibroblasts, and Z-score of LGR5, PROM1, BMI1, ABCG2, EPAS1 was higher in C3 AGT + Fibroblasts. Subsequent bubble plots depicting average expression and percent expressed of differentially expressed stemness genes in the four fibroblast subpopulations revealed that CD34 and KLF4 were highly expressed in C1 LINC01133+ Fibroblasts, MYC in C2 FGF7+ Fibroblasts expression, EPAS1 was highly expressed in C3 AGT + Fibroblasts, and CTNNB1 was high average expression but low percent expressed in C0 THBS4+ Fibroblasts (Figure 5(b)). We depicted the distribution of the expression of the four stemness genes (CD34, KLF4, MYC, EPAS1), which were highly expressed in fibroblast subpopulations, among fibroblast subpopulations by UMAP plots (Figure 5(c)). Meanwhile, the differences in the expression levels of CD34, KLF4, MYC, EPAS1 among the four fibroblast subpopulations were compared using Bar graphs, the results of which were consistent with the heatmap and bubble plot results described above (Figure 5(e)). In addition to the gene expression amounts, we also depicted the density distribution of the four stemness genes in the four fibroblast subpopulation species using UMAP plots (Figure 5(d)). By combining the distribution of fibroblast subpopulations in Figure 2(d), CD34 was denser in C0 THBS4+ Fibroblasts and C1 LINC01133+ Fibroblasts, KLF4 was denser in C1 LINC01133+ Fibroblasts, MYC was denser in C1 LINC01133+ Fibroblasts, C2 FGF7+ Fibroblasts and C3 AGT + Fibroblasts were all denser, and EPAS1 was denser in C0 THBS4+ Fibroblasts and C3 AGT + Fibroblasts.

### 3.6. Differential Expression of Stem Genes in Cardiomyopathy Groups

**Figure 6:**
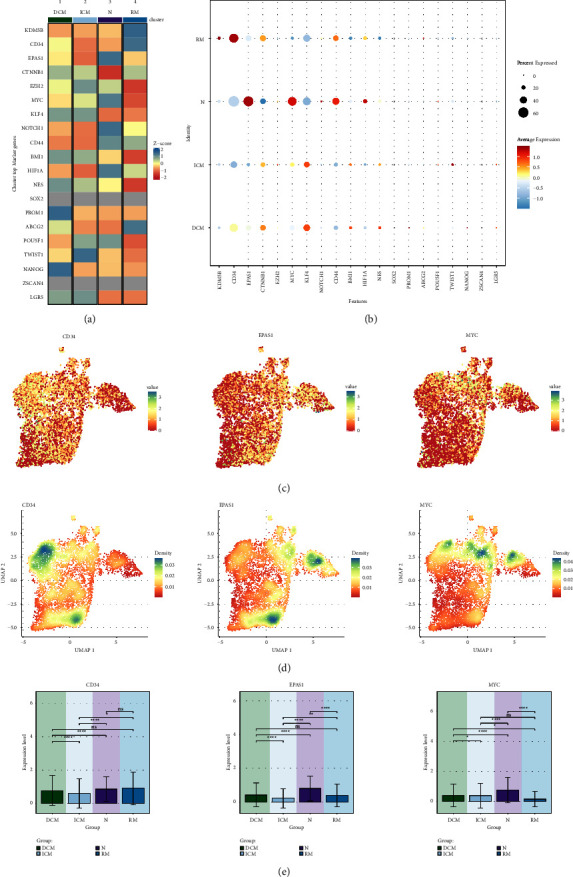
Visualization of expression differences of stemness genes in different groups. (a) Heatmap demonstrated the Z-score of differential stemness genes in 4 groups. (b) Bubble plot demonstrated the average expression level of differential stemness genes of the 4 groups. (c) UMAP plot demonstrated the distribution of the expression of the 3 stemness genes CD34, EPAS1, MYC. (d) UMAP plot showed the distribution of the densities of the 3 stemness genes CD34, EPAS1, MYC. (e) Bar graph demonstrated the comparison of the expression levels of the 3 stemness genes CD34, EPAS1, MYC in the 4 groups. ^*∗*^*p* < 0.05, ^*∗∗*^*p* < 0.01, ^*∗∗∗*^*p* < 0.001, ^*∗∗∗∗*^*p* < 0.0001, ns indicates no statistical difference.

We also noted the same differential expression of stemness genes across different types of cardiomyopathies, with the heatmap showing the top marker genes differentially expressed by the 4 different types of cardiomyopathies and their Z-scores (Figure 6(a)). Among them, the Z-scores of PROM1 and NANOG were higher in DCM, the Z-score of TWIST1 was higher in ICM, the Z-scores of NOTCH1, HIF1A, EPAS1, MYC and CD44 were higher in N, and the Z-scores of KDM5B, CD34 and ABCG2 were higher in RM. Next, we similarly used bubble plots to show the average expression and percent expressed of the differentially expressed stemness genes in the four groups (Figure 6(b)). Average expression and percent expressed of CD34 were both higher in RM, and EPAS1 and MYC's average expression and percent expressed were both higher in N. Therefore, we selected the highly expressed CD34, EPAS1 and MYC for further analysis and demonstrated the distribution of their expression with UMAP plots (Figure 6(c)). And we compared the expression differences of CD34, EPAS1, MYC in the four groups with Bar graph, and learned that CD34 was expressed at higher levels in RM, and EPAS1 and MYC were expressed at higher levels in N (Figure 6(e)), and the results were consistent with Figure 5(b). Finally, in combination with the distribution of fibroblast subpopulations in Figure 2(d), CD34 was more densely expressed in C0 THBS4+ Fibroblasts and C1 LINC01133+ Fibroblasts, EPAS1 was more densely expressed in C0 THBS4+ Fibroblasts and C3 AGT + Fibroblasts, and MYC was more densely expressed in C1 LINC01133+ Fibroblasts, MYC was denser in C2 FGF7+ Fibroblasts and C3 AGT + Fibroblasts (Figure 6(d)).

### 3.7. Gene Regulatory Network Analysis of Cardiomyopathy Fibroblast Subpopulations

**Figure 7:**
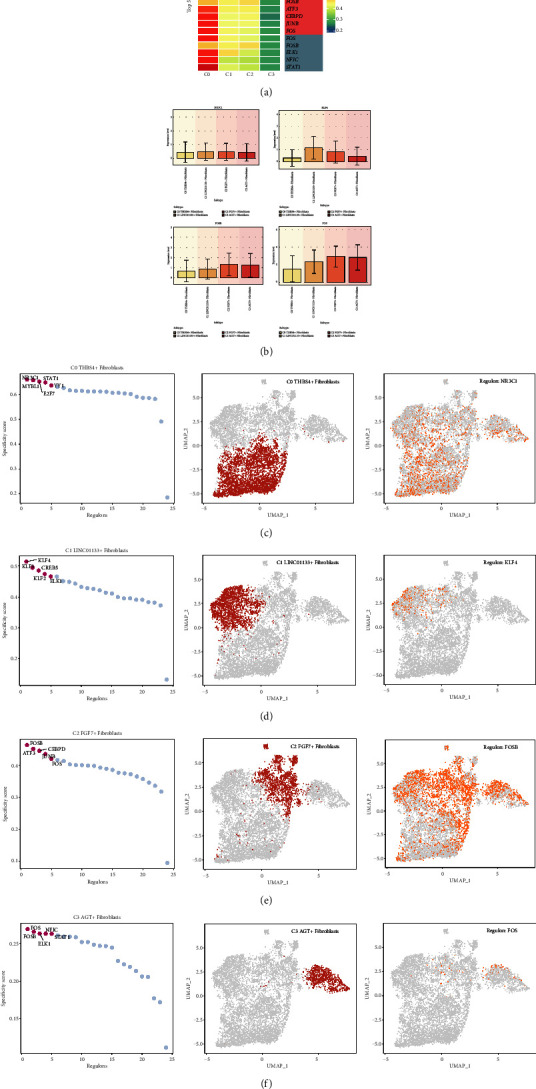
Gene regulatory network analysis of cardiomyopathy fibroblast subtypes. (a) Heatmap demonstrated the expression levels of Top5 TFs in four fibroblast subpopulations. (b) Bar graph demonstrated the ratio of expression levels of NR3C1, KLF4, FOSB, FOS in fibroblast subpopulations. (c–f) Ranked of regulons in cardiomyopathy fibroblast subpopulations based on regulon specificity score (RSS). Cardiomyopathy fibroblasts were highlighted in UMAP (red dots). Binarized regulon activity score (RAS) of the top regulon of cardiomyopathy fibroblast subpopulations on UMAP (Z-score normalization was performed for all samples and 2.5 was set as the cutoff value to convert to 0 and 1) (orange dots).

To identify core TFs detectable in cardiomyopathy fibroblast subpopulations, we performed SCENIC analysis. PySCENIC was used to infer the gene regulatory networks of all cardiomyopathy fibroblast subpopulations. Based on the findings of cell type-specific regulatory activity, the most active TFs in each of the 4 cardiomyopathy fibroblast subpopulations, including NR3C1 (C0 THBS4+ Fibroblasts), KLF4 (C1 LINC01133+ Fibroblasts), FOSB (C2 FGF7+ Fibroblasts), FOS (C3 AGT + Fibroblasts) (Figure 7). Heatmaps were used to demonstrate the differential expression of Top5 TFs in the four fibroblast subpopulations (Figure 7(a)). To visualize the gene expression more, we also used Bar graphs (Figure 7(b)). Regulators in cardiomyopathy fibroblast subpopulations were ranked according to specificity score (RSS). In UMAP, cardiomyopathy fibroblast subpopulations are highlighted (red dots), along with the UMAP-based binarized regulator activity score (RAS) for the major regulators of cardiomyopathy fibroblast subpopulations (Z-score normalized for all samples and converted to 0 and 1 using 2.5 as the cutoff value) (orange dots) (Figures 7(c)–7(f)).

### 3.8. Gene Regulatory Network Analysis of Cardiomyopathy Groups

**Figure 8:**
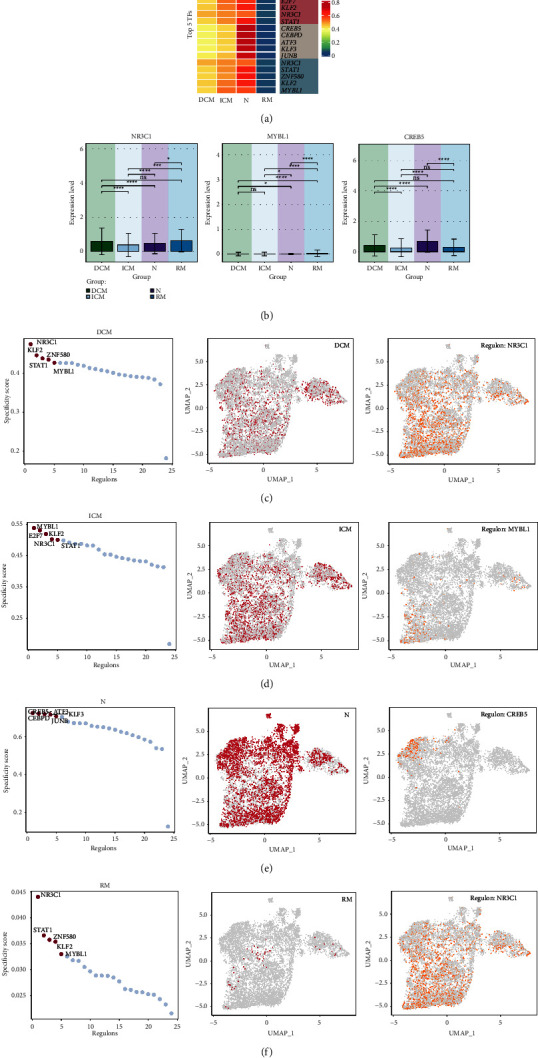
Cardiomyopathy groups gene regulatory network analysis. (a) Heatmap demonstrated the expression levels of Top5 TFs of the 4 groups. (b) Bar graph demonstrated the proportion of expression level of NR3C1, MYBL1, CREB5 in groups. ^*∗*^*p* < 0.05, ^*∗∗*^*p* < 0.01, ^*∗∗∗*^*p* < 0.001, ^*∗∗∗∗*^*p* < 0.0001, ns indicates no statistical difference. (c–f) Ranked of regulons in cardiomyopathy groups based on regulon specificity score (RSS). Cardiomyopathy groups were highlighted in UMAP (red dots). Binarized regulon activity score (RAS) of the top regulon of cardiomyopathy groups on UMAP (Z-score normalization was performed for all samples and 2.5 was set as the cutoff value to convert to 0 and 1) (orange dots).

Similarly, we performed SCENIC analysis in order to identify core TFs detectable in different groups of cardiomyopathy. PySCENIC was used to infer the gene regulatory network of all cardiomyopathy groups. Based on the findings of specific regulatory activities of different groups, the most active TFs in each of the 4 groups, including NR3C1(DCM), MYBL1(ICM), CREB5(N), NR3C1(RM) (Figure 8), with NR3C1 being the most highly expressed in both DCM and RM. Heatmaps were used to show the differential expression of Top5 TFs in the four groups (Figure 8(a)). To visualize the gene expression more, we also used Bar graphs (Figure 8(b)). Regulators in cardiomyopathy groups were sorted according to the specificity score (RSS). In UMAP, cardiomyopathy groups are highlighted (red dots), and the binarized regulator activity scores (RAS) based on UMAP for the main regulators of the four cardiomyopathy groups (Z-score normalized for all samples and converted to 0 and 1 using 2.5 as the cutoff value) are also shown (orange dots) (Figures 8(c)–8(f)).

### 3.9. Identification of TF Regulatory Modules in Cardiomyopathy Fibroblasts and Correlation of Transcriptional Regulatory Activity among Different Subpopulations

**Figure 9:**
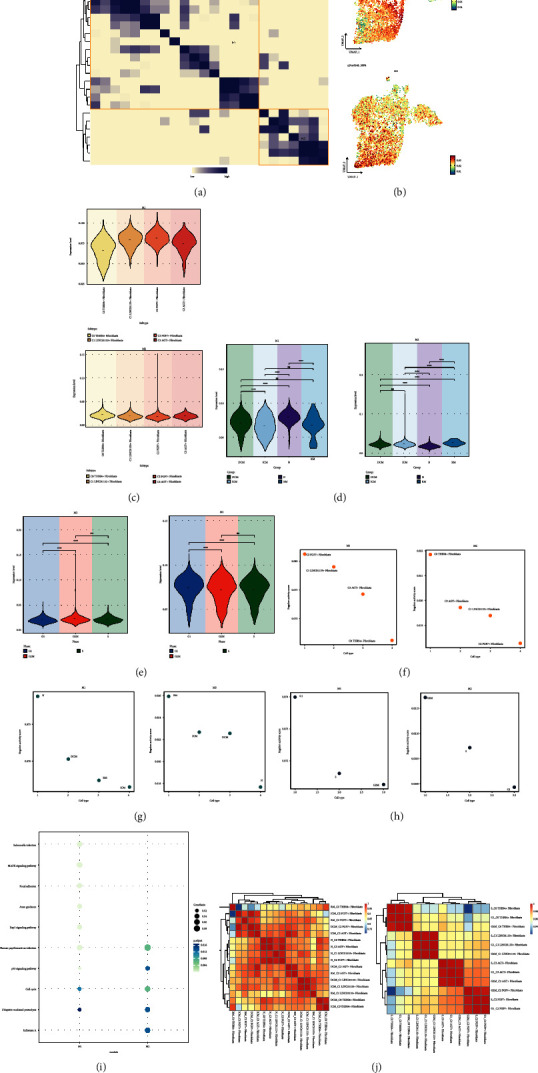
Identification of TFs regulatory modules in cardiomyopathic fibroblasts. (a) Heatmap showed the similarity of different rules based on AUCell scores using the SCENIC identification rule module. 2 rule submodules identified based on rule similarity. (b) UMAP plot showed the distribution of mean AUCell scores for 2 rule submodules (M1, M2). (c) Violin plot demonstrated the proportion of cardiomyopathy fibroblast subpopulations in M1, M2. (d) Violin plot showed the proportion of different groups in M1, M2. (e) Violin plot showed the proportion of different cell cycles in M1, M2. ^*∗*^*p* < 0.05, ^*∗∗*^*p* < 0.01, ^*∗∗∗*^*p* < 0.001, ^*∗∗∗∗*^*p* < 0.0001, ns indicates no statistical difference. (f–h) Scatter plots demonstrated regulon activity score of each fibroblast subpopulation, each group and each cell cycle in MI, M2. (i) Bubble plots demonstrated the enrichment results of all differential genes in M1, M2. (j) Heatmap demonstrated the correlation of transcriptional regulatory activity between fibroblast subpopulations of different groups (left) and between fibroblast subpopulations of different cell cycles (right).

We used the SCENIC identification rule and discovered the regulatory modules of cardiomyopathy fibroblast subpopulations using the connection specific index (CSI) matrix. The similarity of different rules based on AUCell score was categorized into the following two main modules (M1, M2) (Figure 9(a)). Then, UMAP plots further demonstrated that the functions of these TFs were highly specialized to the corresponding one or several cardiomyopathic fibroblast subpopulations (Figure 9(b)). When we mapped the average activity scores of each module onto UMAP, we found that each module occupied a different cellular subpopulation. Combined with Figure2(d), C1 LINC01133+ Fibroblasts, C2 FGF7+ Fibroblasts, and C3 AGT + Fibroblasts were predominantly distributed in M1, and C0 THBS4+ Fibroblasts were predominantly distributed in M2. In order to more visually demonstrate the expression occupancy of the various fibroblast subpopulations in M1 and M2 ratio, we applied a violin plot (Figure 9(c)). In M1, the expression of C1 LINC01133+ Fibroblasts, C2 FGF7+ Fibroblasts and C3 AGT + Fibroblasts was relatively high. In M2, the expression of C0 THBS4+ Fibroblasts was slightly higher than the other subpopulations. Consistent with the results demonstrated by the UMAP plot. In order to understand the expression levels of the four groups and different cell cycles in the two modules of M1, M2, we also applied the intuitive violin plots (Figure 9(d) and 9(e)). The expression levels of N and DCM were higher in M1, the expression level of G1 was higher than the other two periods, and the expression levels of G2M and S were similar and high. The expression levels of RM, DCM and ICM were relatively a little higher in M2. The expression levels of G2M were slightly higher than the other two periods, and the expression levels of G1 and S were similar and low. In addition, we also compared the regulon activity scores of different fibroblast subpopulations, different groups and different cell cycles in M1 and M2 (Figure 9(f)–9(h)). The regulon activity score of C2 FGF7+ Fibroblasts was the highest in M1, and that of C0 THBS4+ Fibroblasts was the lowest, and the regulon activity score of C0 THBS4+ Fibroblasts was the highest and C2 FGF7+ Fibroblasts was the lowest in M2. regulon activity scores of N were the highest and ICM were the lowest in M1, and RM in M2 had the regulon activity score was highest in M1 and lowest in N. Regulon activity score was highest in M1 for G1 phase and lowest for G2M phase, and highest in M2 for G2M phase and lowest for G1.

Next, we enriched all the genes in M1 and M2 and analyzed them, which were presented in bubble plots (Figure 9(i)). The genes in M1 were mainly enriched in Ubiquitin mediated proteolysis and Cell cycle. The genes in M2 were mainly enriched in Ubiquitin mediated proteolysis, Influenza A, p53 signaling pathway, Human papillomavirus infection and Cell cycle. Finally, in order to understand the correlation of transcriptional regulatory activities among different subpopulations of cardiomyopathic fibroblasts, we each divided into subpopulations of fibroblasts with different groups and subpopulations of fibroblasts with different cell cycles. subpopulations of fibroblasts with different cell cycles (Figure 9(j)). We learned from the heatmap that the correlations between ICM_C2 and DCM_C2, N_C0 and N_C3, N_C0 and N_C2, N_C3 and N_C2, DCM_C3 and RM_C3, DCM_C1 and ICM_C1, and DCM_C0 and ICM_C0 were all high. In addition, fibroblasts in each fibroblast subpopulation that were in different cell cycles were highly correlated with each other.

## 4. Discussion

Cardiomyopathy is usually considered to occur at any age and is one of the common dangerous cardiovascular diseases. The presence of myocardial hypoxia in the majority of cardiomyopathies is well established and this is associated with the development of myocardial fibrosis [40]. In the cardiovascular system, myocardial fibrosis originates in nonischemic cardiomyopathies [41]. In ischemic cardiomyopathy (DCM), cardiac fibrosis can drive the progression of heart failure [42]. Therefore, myocardial fibrosis is a serious adverse outcome of cardiomyopathy. And fibroblasts in cardiomyopathy determine the extent and quality of myocardial fibrosis [20]. Proliferation of fibroblasts and cell-specific activation of genetic programs associated with cell migration are factors in the progression of cardiomyopathy [43]. A study suggests that persistent fibroblast efflorescence in patients with long-term heart disease can be therapeutically modulated by targeted silencing in human myofibroblasts [42]. However the intrinsic cellular landscape of relevant pathways among different subtypes of cardiomyopathy fibroblasts in terms of stemness gene expression, metabolic pathway salience, and transcription factor regulatory activity remains unclear. Therefore, we performed visualization and landscape analysis of cardiomyopathy fibroblasts.

First, we used scRNA-seq data from cardiomyopathy. After classification of 32 clusters, removal of batch effects, and initial cell type annotation, 179,927 cells were classified into 11 cell types. Fibroblasts had high proliferation and activity, as they were higher in terms of both nCount_RNA and nFeature_RNA. To demonstrate that the heterogeneity of all cells in cardiomyopathy is equally high, we showed Top5 marker genes for the screened cell types, clarifying the differential expression of specific marker genes in different cell types. Then we performed GO_BP enrichment analysis of different cell types and found that fibroblasts were mainly enriched in the following biological processes: vasculature development, tube development, blood vessel development, circulatory system development, tube morphogenesis, anatomical structure formation involved in morphogenesis, system process. These enrichment pathways are all related to the formation and development of anatomical structures such as blood vessels and the development and operation of the circulatory system, proving that fibroblasts are mainly enriched in functioning, demonstrating that fibroblasts are intimately involved in the structure of the myocardium and may contribute in part to the progression of myocardial fibrosis in cardiomyopathy. To further understand the functional status of all genes among fibroblasts, we enriched them again and found that genes in fibroblasts have important roles in activation of immune responses and spatial transport of proteins and enzymes.

To reveal the heterogeneity among different subpopulations of fibroblasts, we further divided the 8148 cardiomyopathic fibroblasts into 4 subpopulations (C0 THBS4+ Fibroblasts, C1 LINC01133+ Fibroblasts, C2 FGF7+ Fibroblasts, C3 AGT + Fibroblasts). Each subpopulation was named after its top marker. Among them, C3 AGT + Fibroblasts had the highest percentage of ischemic cardiomyopathy (RM), and coincidentally ICM had a worse prognosis. Therefore, we inferred that C3 AGT + Fibroblasts are more sensitive and active than other fibroblast subpopulations for the progression of cardiomyopathy as well as fibrosis. In addition, we also found that C3 AGT + Fibroblasts were located at the end stage of the pseudo-temporal sequence with the highest degree of differentiation by pseudo-temporal sequencing. There are also the results of enrichment pathway by GO_BP enrichment analysis and all the differential genes of this subgroup, all three of which indicate that C3 AGT + Fibroblasts are more active for the progression of cardiomyopathy and myocardial fibrosis. In addition, the GO_BP enrichment results also showed that the enrichment score of the biological process of muscle contraction were higher in C3 AGT + Fibroblasts than in other cardiomyopathy fibroblast subpopulations. This demonstrated that myocardial contractile dysfunction in cardiomyopathy may be associated with fibroblasts. In addition, a significant etiological factor of dilated cardiomyopathy (DCM) is oxidative stress, characterized by an imbalance between the accumulation of reactive oxygen species (ROS) and the body's antioxidant defense mechanisms [44]. Excessive ROS accumulation can lead to sustained loss of potassium ions and high-energy phosphates, as well as an increase in cytosolic calcium ion concentration, resulting in decreased contractile force of myocardial cells. The key factors in ROS generation are related to metabolic and mitochondrial respiratory chain dysfunction, particularly oxidative phosphorylation [45].Therefore, through the enrichment of fibroblast subsets in processes like oxidative phosphorylation, we inferred that certain types of cardiomyopathy might have been driven by abnormal activity levels of fibroblasts. We believed that this held potential significance for the diagnosis of certain types of cardiomyopathy, which required further validation.

Meanwhile, the above results from AUCell on the metabolism of a subset of cardiomyopathic fibroblasts indicate that Oxidative phosphorylation is a major metabolic pathway in fibroblasts, and that it scores particularly high in C1 LINC01133+ Fibroblasts and C3 AGT + Fibroblasts. Oxidative phosphorylation is mainly the process of driving ATP by the energy released from the oxidative step in the catabolism of organic substances, including sugars, lipids, and amino acids. This result likewise demonstrates that ATP production is higher in C3 AGT + Fibroblasts, with richer biological processes and more prominent effects on disease development. As mentioned above, in C3 AGT + Fibroblasts, oxidative phosphorylation increases the production of reactive oxygen species (ROS), ultimately leading to a decrease in the contractile force of myocardial cells. Besides, Glycolysis/Gluconeogenesis activity was also more significant in C3 AGT + Fibroblasts. Glycolysis is not dependent on oxygen and produces two molecules of ATP in its process, so under hypoxic conditions (low oxygen), the rate of Glycolysis increases to compensate for the reduced oxidative respiration to meet the cell's energy requirements. In contrast, the primary physiological significance of Gluconeogenesis is to ensure a relatively constant blood glucose concentration in the presence of starvation. Therefore, we hypothesized that C3 AGT + Fibroblasts may have the ability to overcome difficult environments (e.g., hypoxia and starvation) and thus continue to influence disease progression.

Stemness refers to the ability to self-renew and differentiate into mature cells. Therefore, we performed the same study on stemness genes and found that the expression of stemness genes is also heterogeneous in cardiomyopathic fibroblasts. Four stemness genes, CD34, KLF4, MYC, and EPAS1, which were highly labeled among subpopulations of fibroblasts, were screened by comparing their Z scores, average expression, and percent expressed. Among them, EPAS1 was highly expressed in C3 AGT + Fibroblasts. In addition, we visualized the density distribution of these four stemness genes by mapping to UMAP. We found that MYC was denser in C1 LINC01133+ Fibroblasts, C2 FGF7+ Fibroblasts, and C3 AGT + Fibroblasts, but was only highly expressed in C2 FGF7+ Fibroblasts.EPAS1 was highly expressed in C0 THBS4+ Fibroblasts and C3 AGT + Fibroblasts was denser in C0 THBS4+ Fibroblasts and C3 AGT + Fibroblasts, but was only highly expressed in C3 AGT + Fibroblasts. We then proceeded to compare the expression of stemness genes in different groups of cardiomyopathies using the same method, and screened for the same high expression of CD34, EPAS1, and MYC. the results showed that EPAS1 and MYC had high expression in N, and we hypothesized that EPAS1 and MYC had a possible correlation with the improvement of cardiomyopathy. Meanwhile, according to previous literature, EPAS1, an endothelial PAS domain protein 1, has been found to have some association with the stability of HCM [46]; MYC is one of the top ten hub genes in diabetic cardiomyopathy (DbCM) [47]. FOS has been shown to be associated with heart development, having a certain correlation with DCM, and upregulated in protective cells, potentially involved in regulating the differentiation fate of cardiac fibroblasts under pressure overload [48, 49]. Because EPAS1 was densely and highly expressed in C3 AGT + Fibroblasts, and MYC was densely but poorly expressed in C3 AGT + Fibroblasts, whether inhibition or promotion of EPAS1 and MYC is beneficial to the treatment of cardiomyopathy requires further study.

To explore the key TFs among cardiomyopathy fibroblast subpopulations, we employed gene regulatory network analysis for further study. Based on the CSI results, two major modules, M1, and M2, were identified among cardiomyopathy fibroblast subpopulations. In addition, we found that the fibroblast subpopulations in M1 were mainly C1 LINC01133+ Fibroblasts, C2 FGF7+ Fibroblasts and C3 AGT + Fibroblasts, and C0 THBS4+ Fibroblasts were predominantly found in M2.We also firmly established that cardiomyopathic fibroblasts subpopulations and different groups of key TFs, including NR3C1 (C0 THBS4+ Fibroblasts), KLF4 (C1 LINC01133+ Fibroblasts), FOSB (C2 FGF7+ Fibroblasts), FOS (C3 AGT + Fibroblasts), NR3C1 (DCM) FOS(C3 AGT + Fibroblasts), MYBL1(ICM), CREB5(N), NR3C1(RM).FOS is the most important TF of C3 AGT + Fibroblasts and is associated with the M1 module. The regulon activity score of N is the highest in M1, and the highest in G1 phase, and the lowest in G2M phase. Meanwhile the major genes in M1 were mainly enriched in Ubiquitin mediated proteolysis and Cell cycle. Therefore, we hypothesized that FOS may influence with the development of cardiomyopathy through protein hydrolysis and cell cycle progression.

Finally, we further elucidated the correlation of transcriptional regulatory activities among different subpopulations of cardiomyopathic fibroblasts and found that C3 AGT + Fibroblasts in N were highly correlated with C0 THBS4+ Fibroblasts and C2 FGF7+ Fibroblasts that were also in N. C3 AGT + Fibroblasts in DCM and C3 AGT + Fibroblasts in RM were highly correlated. And within each fibroblast subpopulation, they were highly correlated even among cells in different cell cycles. Thus, C3 AGT + Fibroblasts in the normal group had a modest correlation with other subpopulations, whereas C3 AGT + Fibroblasts after the development of cardiomyopathy lost some correlation with other subpopulations. We hypothesized that the heterogeneity of C3 AGT + Fibroblasts in cardiomyopathy is more prominent, and the possibility exists with the development of cardiomyopathy.

## 5. Conclusion

Based on the single-cell characterization of cardiomyopathic fibroblasts, we conclude that C3 AGT + Fibroblasts may be more sensitive to cardiomyopathy, whereas the activity of other subpopulations may be more suppressed. Differences in stemness gene expression and differences in transcriptional regulatory activity between C3 AGT + Fibroblasts and other subpopulations of cardiomyopathic fibroblasts may provide a novel perspective that the differential gene expression, stemness differences, and transcriptional regulator differences in C3 AGT + Fibroblasts may influence cardiomyopathy outcomes such as myocardial fibrosis. This provides a direction for improving the unfavorable prognosis of cardiomyopathy. If possible, it also offers some references for potential therapeutic targets in the treatment of cardiomyopathy. More importantly, the stemness genes EPAS1, MYC, and the regulator FOS with C3 AGT + Fibroblasts may regulate the biological processes of cardiomyopathic fibroblasts through the corresponding pathways, thereby affecting disease progression. Although our bioinformatics analyses have yielded insights of valuable significance, we lack specific experimental validation, which limits further justification of the conclusions presented in our study. Therefore, in order to gain a clearer understanding of the specific mechanisms of influence and outcomes, we need to further advance our research.

## Data Availability

The datasets of single-cell and bulk sequencing generated and/or analyzed during the current study are publicly available in the GEO. The inquiries of original contributions presented in the study can be directed to the corresponding authors.
